# Randomized clinical evaluation of self-screening for anal cancer precursors in men who have sex with men

**DOI:** 10.1186/1742-6413-3-4

**Published:** 2006-03-20

**Authors:** Thomas M Lampinen, Mary Lou Miller, Keith Chan, Aranka Anema, Dirk van Niekerk, Arn J Schilder, Robert Taylor, Robert S Hogg

**Affiliations:** 1 BC Centre for Excellence in HIV/AIDS, Vancouver, British Columbia, Canada; 2 Department of Health Care and Epidemiology, University of British Columbia, Vancouver, Canada; 3 BC Cancer Agency, Vancouver, Canada; 4 St. Paul's Hospital, Anal Dysplasia Clinic, Vancouver, Canada

## Abstract

**Background:**

Self-collection of anorectal swab specimens could greatly facilitate the completion of prerequisite studies and future implementation of anal cancer screening among men who have sex with men (MSM). We therefore compared self- versus clinician- collection procedures with respect to specimen adequacy for cytological evaluation, concordance of paired cytological results, and concordance of cytological with biopsy results.

**Methods:**

Paired self- and clinician- collected anorectal Dacron^® ^swabs for liquid-based (Thin Prep^®^) cytological evaluation were collected in random sequence from a mostly HIV-1 seronegative cohort of young MSM in Vancouver. Slides were reviewed by one cytopathologist. Presence of any cytological abnormality (atypical squamous cells of uncertain significance, ASCUS, or above) prompted referral for high-resolution anoscopy and possible biopsy.

**Results:**

Among 222 patient-clinician specimen pairs, most were adequate for cytological evaluation, though self-collected specimens were less likely to be so (83% versus 92%, McNemar's test p < 0.001). Cytological abnormalities, noted in 47 (21%) of self-collected and 47 (21%) of clinician-collected specimens (with fair agreement, kappa = 0.414) included, respectively: ASCUS (5%, 5%), and low-grade (13%, 13%) and high-grade (3%, 3%) squamous intraepithelial lesions. Among 12 men with biopsy-confirmed high-grade neoplasia, most had abnormal cytological results (including 6 patient and 9 clinician swabs) but few (2 patient and 1 clinician swab) were high-grade.

**Conclusion:**

Self-collection of anorectal swab specimens for cytologic screening in research and possibly clinical settings appears feasible, particularly if specimen adequacy can be further improved. The severity of biopsy-confirmed anorectal disease is seriously underestimated by cytological screening, regardless of collector.

## Introduction

Anal cancer in the general population is rare, with an incidence of only 0.8 cases per 100,000 per year. However, the incidence of this malignancy has increased during the past three decades [1-3]. Risk of anal cancer is greatly elevated among HIV-1 seronegative and HIV-1 seropositive men who have sex with men (MSM), in whom 35-fold and 70-fold relative risks, respectively, have been reported [4-6]. Since publication of these risk estimates, two very recent studies suggest that incidence rates among HIV-1 seropositive MSM may have further doubled, owing to increased survival since 1996 among men receiving potent antiretroviral treatment [7,8].

Among MSM, the prevalence of human papillomavirus (HPV) infection and of precursor anal squamous intraepithelial lesions does not appear to decrease with age [9,10]. Early detection and ablation of precursor lesions in the anal canal might reduce mortality from anal cancer, just as early detection by Pap smear and treatment has reduced mortality from cervical cancer [11]. Liquid-based anal cytological specimens perform comparably to conventional anorectal smears [12] and though modest in sensitivity, both may be adequate for screening purposes [12-17].

However, estimates of the cost-effectiveness of screening are strongly sensitive to model assumptions about the rate with which high-grade squamous intraepithelial lesions progress to invasive anal cancer, a rate that is unknown, and to the effectiveness of treatment in preventing this progression [18,19]. Current treatment of HIV-1 seronegative MSM appears to be effective in clearing abnormal squamous cells from the anorectal canal; in contrast, an extraordinary rate of anogenital lesion persistence and recurrence among HIV-1 seropositive men and women represents a serious impediment to implementing routine screening at this time [20-22].

Development of evidence-based policies regarding routine screening of MSM for anal cancer therefore requires collection of additional natural history, treatment and acceptability data from large, diverse samples of men [23,24]. These studies will be easier and less costly to complete if anorectal swab specimens for cytological evaluation can be self-collected. Future screening coverage is also likely to be greater if self-sampling is possible. Only one evaluation of such self-collection has been published and that study included only 20 HIV-1 seronegative MSM [25]. We therefore compared self- versus clinician-collection procedures in a large cohort of mostly HIV-1 seronegative MSM; our endpoints included specimen adequacy for cytological evaluation, concordance of cytological results, and relative concordance of cytological and histopathological results.

## Methods

### Study population

We conducted a cross-sectional study within the Vanguard Project, a prospective cohort of HIV incidence and risk behaviors among young HIV-1 seronegative MSM aged 18 to 30 in Vancouver [26,27]. The study protocol was approved by the University of British Columbia Clinical Ethics Research Board. Each participant provided written informed consent and received a $20 honorarium. Study visits included completion of a brief self-administered questionnaire.

### Specimen collection

Each participant provided at the same study visit two anorectal swab specimens: one self-collected and one collected by the study clinician. Because we were unsure whether the second swab would yield fewer cells and be less adequate for cytological evaluation, we randomly assigned each individual the order in which their paired swabs (self versus clinician) were to be collected. The order was designated on a card placed in a sealed envelope and included in previously prepared specimen collection kits. These kits included two sterile Dacron^® ^polyester tipped swabs, two bottles of PreservCyt^® ^solution (Cytyc Corporation, Boxborough Mass.), a biohazard transport bag, and illustrated step-by-step instructions for self-collection [28].

Swabs were inserted approximately 4–6 cm beyond the anal verge, then used to gently wipe the anal canal wall, then rotated 360° during slow removal. The swab was then placed in PreservCyt^®^, the plastic shaft was snapped, and the container was capped tightly. Self-and clinician-collected specimens were labeled with unique identification numbers, placed in separate transport bags, and sent immediately for cytological examination.

### Cytological examination

One cytopathologist (DvN), reviewed all slides without knowledge of their having been self- or clinician- collected. Samples were considered adequate for cytological examination regardless of the presence of glandular cells, as long as they contained 5,000 well-preserved squamous cells, at least 75% of which were not obscured by covering inflammation, blood or fecal material. Adequate specimens were evaluated using 2001 Bethesda criteria and diagnosed as moderate to high-grade squamous intraepithelial lesion (high-grade, or HSIL), low-grade squamous intraepithelial lesion (low grade, or LSIL), atypical squamous cells of undetermined significance (ASCUS), or negative for atypical or malignant cells ("normal"). Men with any cytological abnormality (including ASCUS) were referred promptly for anoscopy and possible biopsy.

### Statistical analysis

Group comparisons were performed using two-sided Chi-square, Fisher's Exact, and Wilcoxon rank sum tests (alpha = 0.05). Pair-wise agreement with respect to binary endpoints (specimen adequacy, detection of any cytological abnormality including ASCUS) were performed using McNemar's Chi-square and kappa statistics computed with SAS v.8.2 for PC (SAS Institute, Cary, NC).

## Results

Between July 2003 and April 2004, 222 men provided paired anorectal swab specimens for cytological examination. The median age of study participants was 31.5 years and approximately half had completed college (Table 1). Most men were HIV-1 seronegative; the 28 HIV-1 seroconverters had been infected for a median of 2.0 years. No significant differences among measured demographic characteristics were observed between men randomly assigned to initial self-collection versus clinician collection (Table 1).

**Table 1:** Characteristics of study participants, overall and by randomly assigned order of swab collection.

	**Clinician Collected First Specimen (n = 115)**	**Client Collected First Specimen (n = 107)**	**Total Study Population (n = 222)**
Age, median years (IQR)	31.0 (28, 35)	32.0 (28, 35)	31.5 (28, 35)
College graduate, n (%)			
Yes	50 (45)	51 (50)	101 (47)
No	61 (55)	51 (50)	112 (53)
Full-time employment, n (%)			
Yes	56(50)	62 (61)	118 (55)
No	57 (50)	41 (39)	98 (45)
Stable housing^a^, n (%)			
Yes	99 (86)	93 (88)	192 (87)
No	16 (14)	13 (12)	29 (13)
Sex trade involvement^a^, n (%)			
Yes	13 (12)	11 (11)	24 (11)
No	100 (89)	93 (89)	193 (89)
Injection drug use^a^, n (%)			
Yes	12 (11)	7 (7)	19 (9)
No	101 (89)	98 (93)	199 (91)
HIV-1 seropositive, n (%)			
Yes	18 (16)	10 (9)	28 (13)
No	97 (84)	97 (91)	194 (87)

^a ^in previous 12 months

### Cytological adequacy of specimens

Specimens collected second were not cytologically less adequate than those collected first, despite significant pair-wise disagreement for this endpoint (McNemar's test p = 0.040, kappa = 0.288). Among the 222 participants, 44 had at least one anorectal swab specimen inadequate for cytological evaluation: in 10 men (4.5%) both specimens were inadequate, in 23 men (10.4%) only the first swab was inadequate, and in 11 men (5.0%) only the second swab was inadequate (Table 2).

**Table 2:** Paired cytology results, by order of anorectal swab collection (n = 222).

**Second Swab Result**	**First Swab Result**					
	
	Normal	ASCUS	LSIL	HSIL	Inadequate	Total
Normal	114	4	12	5	21	156 (70)
ASCUS	10	0	1	2	1	14 (6)
LSIL	10	3	13	0	1	27 (12)
HSIL	0	0	2	2	0	4 (2)
Inadequate	6	3	2	0	10	21 (9)
Total	140 (63)	10 (5)	30 (14)	9 (4)	33 (15)	222 (100)

Similarly, specimens collected second were not less likely to have a cytological abnormality (ASCUS, LSIL or HSIL) detected, compared to those collected first (McNemar's test p = 0.564, kappa = 0.353). Cytological abnormalities were detected in a total of 71 men: both specimens were abnormal in 23 men (10.4%), the first swab alone was abnormal in 26 men (11.7%), and the second swab alone was abnormal in 22 men (9.9%) (Table 2).

Overall, the number of cytologically inadequate specimens was low among self-collected swabs (n = 37, 16.7%) and among clinician-collected swabs (n = 17, 7.7%). However, we found self-collected specimens were less frequently adequate, compared to clinician-collected specimens (McNemar's test p<0.001, kappa = 0.297). In addition to 10 men (4.5%) in whom both swabs were inadequate, 27 men (12.2%) had an inadequate self-collected swab specimen, and 7 men (3.2%) had an inadequate clinician-collected swab specimen (Table 3).

**Table 3:** Paired cytology results, by collector of anorectal swab specimen (n= 222).

**Clinician-Collected Specimen**	**Self-Collected Specimen**					
	
	Normal	ASCUS	LSIL	HSIL	Inadequate	Total n (%)
Normal	114	7	13	3	21	158 (71)
ASCUS	7	0	1	1	3	12 (5)
LSIL	9	3	13	1	3	29 (13)
HSIL	2	1	1	2	0	6 (3)
Inadequate	6	1	0	0	10	17 (8)
Total n (%)	138 (62)	12 (5)	28 (13)	7 (3)	37 (17)	222 (100)

We did not find that specimens self-collected by men already experienced in the procedure as performed by a clinician (i.e., self-collected second swabs) were more adequate that those collected by men wholly naïve to the procedure (i.e., self-collected first swabs) [21 (19.6%) of 107 versus 16 (13.9%) of 115 respectively, Chi-square p = 0.253]. Further, in the vast majority of men having inadequate self- [21 (78%) of 27] or clinician-collected [6 (86%) of 7] specimens, the cytological diagnoses available from the alternate swab specimen were normal [21 (7.8%) of 27 and 6 (86%) of 7, respectively]; none were high-grade (Table 3).

### Detection of cytological abnormalities

The overall numbers of self- and clinician-collected swabs with a cytological abnormality [47 (21%) of 222 each] were identical. Most cytologically adequate specimens collected by participants [138 (74.6%) of 185] and the clinician [158 (77.1%)] were normal; these proportions were not significantly different (Chi-square p = 0.568). Although based on a small number of seroconverters in our sample, men with and without HIV-1 infection were not significantly different with respect to detection of a cytological abnormality (Chi-square p = 0.143).

We did not observe significant pair-wise disagreement between self-and clinician-collected swabs with regard to detection of a cytological abnormality (McNemar's test p = 0.435, kappa 0.414): both specimens were abnormal in 23 men (12.9%), the self-collected swab alone was abnormal in 23 men (12.9%) and the clinician-collected swab alone was abnormal in 18 men (13.6%). Despite this fair agreement with regard to the presence of any cytological abnormality, the specific diagnoses obtained in paired self- and clinician-collected specimens differed in many instances (Table 3).

### Correspondence of cytological with histopathological results

Among the 71 men in whom any cytological abnormality was noted, 64 (90.1%) returned for follow-up anoscopic examination and 41 underwent biopsies of visible internal lesions. Histopathological findings in the latter included 11 normal diagnoses, 18 diagnoses of AIN 1, and 12 diagnoses of AIN 2/3 (Table 4).

**Table 4:** Comparison of 64 paired self- and clinician-collected anorectal cytologic results in relation to results from anoscopy and possible biopsy.

		**Biopsy Result**									
		
		**Biopsy Not Performed**					**Normal**				
			
		Patient Cytology Result					Patient Cytology Result				
		Inadequate	Normal	ASCUS	LSIL	HSIL	Inadequate	Normal	ASCUS	LSIL	HSIL
Clinician Cytology Result	Inadequate	2		1			2				
	Normal		2	2	2	1		1		1	
	ASCUS	1	4				1				
	LSIL		2	1	3				1	3	
	HSIL					2					2

		**AIN 1**					**AIN 2/3**				
		
		Patient Cytology Result					Patient Cytology Result				
		
		Inadequate	Normal	ASCUS	LSIL	HSIL	Inadequate	Normal	ASCUS	LSIL	HSIL

Clinician Cytology Result	Inadequate							1			
	Normal		2	2	4				1		1
	ASCUS				1		1	1			
	LSIL	1	2	1	4		2			3	1
	HSIL		1					1			

High-grade anal intraepithelial neoplasia (AIN 2 or AIN 3) was confirmed by biopsy in a total of 12 men. Of note, among these 12 men, few had moderate or high-grade diagnoses (HSIL) in their self-collected (2 of 9 adequate swabs) or clinician-collected (1 of 11 adequate swabs) specimens (Table 4). However, most cytological results for these 12 men (including 6 patient and 9 clinician) were abnormal, using ASCUS or above as our criterion.

## Discussion

In our community-recruited cohort of young, highly educated, mostly HIV-1 seronegative MSM naive to anorectal cytologic sampling, we found the vast majority of self-collected swab specimens were adequate for examination, though self-collected specimens were less likely to be so. Further, we did not observe any material difference in the overall distribution of cytological diagnoses obtained using clinician- versus self-collected specimens. However, we noted only fair within-pair agreement with respect to both the presence of any abnormality and the specific cytological diagnoses in self- and clinician-collected specimens. This latter result was expected, as individuals' anorectal specimens collected over short periods of time display similarly poor concordance. For example, the detection of any abnormality in serial specimens collected over time from MSM in another study yielded a correlation similar to our own (kappa = 0.33) [29]. This limited concordance probably reflects sampling variability and only moderate levels of intra-and inter-observer agreement in the reading of anorectal cytological specimens [30].

We observed considerable discordance between individuals' cytological and histopathological diagnoses. Importantly, anorectal cytology underestimated the severity of neoplasia in nearly every case of biopsy-confirmed high-grade neoplasia, regardless of who collected the swab specimen. As well, we found that a greater number of clinician-collected (n = 9) than self-collected (n = 6) specimens obtained from these 12 men were cytologically abnormal (ASCUS or above) and thus would have prompted referral for follow-up anoscopy and possible biopsy.

Our results are consistent with the only previous study to evaluate cytological specimens self-collected by MSM. Cranston et al reported a nearly identical adequacy (85%) among 20 cytological specimens self-collected by HIV-1 seronegative MSM [25]. In that study, cytological abnormalities were noted in only 4 of 10 men with biopsy-confirmed moderate or high-grade disease. Similarly, we found that only 3 of 12 men with biopsy-confirmed high-grade disease had a corresponding cytological diagnosis; most had cytological diagnoses of low-grade dysplasia or ASCUS. In this regard, our results support the recommendation by others that detection of any cytological abnormality (including ASCUS) in anorectal specimens warrants referral of MSM for high-resolution anoscopy [13,16].

The distribution of cytological and histological diagnoses in both self-collected and clinician-collected specimens in our study is similar to that reported from previous studies that included HIV-1 seronegative MSM [5,25,31,32]. Using any cytological abnormality (including ASCUS) as our criterion for referral, we found one fifth of HIV-1 seronegative men require follow-up anoscopy on the basis of a single screen and that about one third require such referral when two specimens are collected; clearly, repeat testing will improve detection of anorectal disease. Nonetheless, two concerns in particular should give pause to those who would at present advocate routine cytological screening of MSM, including those who are HIV-1 seropositive: (1) the lack of effective treatments and (2) the need to revisit assumptions underlying claims that screening is cost-effective.

The present study has unique strengths and represents the first truly controlled evaluation of self-screening among MSM. Unlike the only other self-collection study involving MSM [25], both of our cytological specimens were liquid-based and collected at the same study visit; the order of swab collection was random; we provided detailed, illustrated instructions for the self-collection procedure [28]; and cytological evaluation by one cytopathologist was performed blind to the source of the specimen. Further, rates of follow-up anoscopy and biopsy were very high. Finally, the MSM we studied were wholly inexperienced with regard to collection of anorectal swabs for cytological evaluation. As with almost all studies of MSM, ours was not a population-based sample and the representativeness of our study participants is unclear; at minimum, our results should not be generalized to HIV-1 seropositive women and men.

Results from the present study have important short- and long-term implications. Their immediate significance pertains to research. We have demonstrated that self-collection is possible and only marginally worse than clinician-collected cytology. However, our finding that more men with biopsy-proven high-grade disease were detected during clinician- than during self-screening underscores the need for more work in this area.

If self-collection performs suitably in follow-up studies that aim to improve specimen adequacy, data needed to inform decisions regarding introduction of routine screening might be generated more rapidly and at reduced cost in large samples of MSM. At present, studies are needed to measure the prevalence of anal squamous intraepithelial lesions in more representative samples of MSM, the rate of spontaneous regression of lesions, the efficacy and effectiveness of treatment, and the acceptability of screening in diverse sub-populations of MSM. Additional studies are also required to correct for verification bias in estimating the sensitivity, specificity and predictive values of cytological screening; these studies require biopsies from large numbers of cytologically normal MSM [33,34].

In summary, we found the vast majority of self-collected anorectal swab specimens from MSM were adequate for cytological evaluation. We also found similar frequencies of abnormalities in self- and clinician-collected specimens. Despite the generally comparable performance of clinician- and self-collected anorectal specimens, an important goal is the improvement of cytological adequacy in the latter. Based on the results of our study, this is unlikely to be achieved by simply having MSM undergo their first screen by a trained clinician; rather, it will probably be necessary that men use a larger Dacron^® ^swab, or self-collect a second swab for placement into the same PreservCyt^® ^container. Studies also need to demonstrate concordance of type-specific HPV and its correlates in self- and clinician-collected specimens; toward this end, initial results using a sub-sample of specimens collected during this study are very promising [35].

## Competing interests

The author(s) declare that they have no competing interests.

## Authors' contributions

TL conceived and designed the study, acquired partial funding, supervised the research team and data analysis by KC, and wrote the report with AA. MLM, DVN and AS assisted with study design and acquisition of data. AA contributed to the interpretation of data. RT assisted with acquisition of data. RH contributed to the study design and acquired funding. All authors were involved in the critical revision of the manuscript drafts and approved the final version for publication.
